# Role of Edge Inclination in an Optical Microdisk Resonator for Label-Free Sensing

**DOI:** 10.3390/s150304796

**Published:** 2015-02-26

**Authors:** Davide Gandolfi, Fernando Ramiro-Manzano, Francisco Javier Aparicio Rebollo, Mher Ghulinyan, Georg Pucker, Lorenzo Pavesi

**Affiliations:** 1 Nanoscience Laboratory, Dept. Physics, University of Trento, Via Sommarive 14, I-38123 Trento, Italy; E-Mails: f.ramiromanzano@unitn.it (F.R.-M.); fjaparicio@icmse.csic.es (F.J.A.R.); lorenzo.pavesi@unitn.it (L.P.); 2 Center for Materials and Microsystems, Fondazione Bruno Kessler, via Sommarive 18,; I-38123 Trento, Italy; E-Mails: ghulinyan@fbk.eu (M.G.); pucker@fbk.eu (G.P.)

**Keywords:** whispering gallery mode, optical resonators, wedge resonator, optical sensing, refractometric sensing, biological sensing, limit of detection, figure of merit

## Abstract

In this paper, we report on the measurement and modeling of enhanced optical refractometric sensors based on whispering gallery modes. The devices under test are optical microresonators made of silicon nitride on silicon oxide, which differ in their sidewall inclination angle. In our approach, these microresonators are vertically coupled to a buried waveguide with the aim of creating integrated and cost-effective devices. Device modeling shows that the optimization of the device is a delicate balance of the resonance quality factor and evanescent field overlap with the surrounding environment to analyze. By numerical simulations, we show that the microdisk thickness is critical to yield a high figure of merit for the sensor and that edge inclination should be kept as high as possible. We also show that bulk-sensing figures of merit as high as 1600 RIU^−1^ (refractive index unit) are feasible.

## 1. Introduction

It was more than a decade ago when a whispering gallery mode (WGM) optical resonator was used to experimentally prove the feasibility of a label-free biosensor [1]. Following up the original proposal [2], Vollmer and colleagues measured the resonance-wavelength shift induced by a layer of proteins on a handcrafted silica microsphere [3]. Since then, many other authors have elaborated this concept, improving its scalability [4,5], the sensitivity of the resonator [6,7], the quality factor (Q) [8,9,10], the integration of the sensors in complex and automated systems [11] and the biological functionalization technique for the specific recognition of the target biomolecule [12].

We recently demonstrated the possibility to integrate on-chip a monolithic free-standing disk resonator with a vertically-coupled bus waveguide [13]. Our further works showed how the vertical coupling architecture is particularly suited to couple integrated high-Q wedge resonators, where the sidewall of the resonator is tilted with respect to the surface of the substrate [14].

A very high Q is a key aspect for the realization of sensors with the highest resolution. In this sense, wedge resonators could be good candidates for the fabrication of high performance integrated sensors. In this paper, we investigate both experimentally and theoretically these structures from the point of view of their application as refractive index sensors.

The experimental observations that motivated this work are reported in the second section. Therein, we experimentally characterize and compare the optical sensing parameters of a wedge resonator to those of a disk resonator with similar dimensions.

Then, the model for analyzing the effects of the shape of the structures under test from a sensor point of view is developed in the third section. Finally, we numerically solve the optimization problem by means of finite element analysis. We find the critical geometrical parameters for achieving the best sensing performances, and we compare the results to that of the measured samples.

## 2. Experimental Observations

Wedge resonators are attractive because of their demonstrated feasibility of obtaining on-chip ultra-high quality factors, which is particularly appealing for many applications [9]. This class of devices is made up of WGM optical resonators lithographically defined by means of an isotropic etching step. Therefore, their sidewalls are not vertical, and the angle with the substrate can be varied to some extent by tuning the photoresist adhesion [9] and etching parameters.

**Figure 1:**
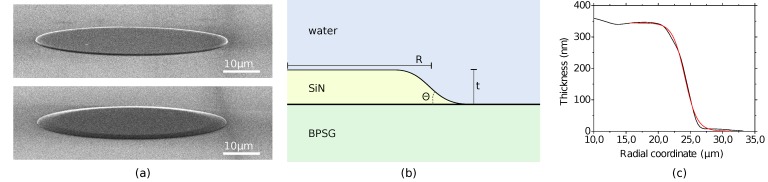
(**a**) SEM micrographs of vertically-coupled monolithically-integrated disk and wedge microresonators. The buried waveguide can be barely seen below the surface. Note the difference in sidewall angle; (**b**) Model of the cross-section profile of the wedge WGM resonator. *R*, *t* and θ are, respectively, the radius, thickness and inclination angle; (**c**) AFM profile of the sidewall of a wedge resonator. The red line is the fitted logistic function (1). Note that the axes’ scales have been modified to properly show the small aspect-ratio of the structure.

In Figure 1a, we compare the SEM micrographs of two WGM resonators obtained with different etching processes. By using an isotropic wet etch in a buffered hydrofluoric acid (BHF) solution, the sidewall angle is clearly reduced, compared to the case of the anisotropic reactive ion etching (RIE).

A simple scheme of a wedge WGM resonator is shown in Figure 1b, with the definitions of radius (*R*), thickness (*t*) and inclination (θ). AFM images of some of our structures (Figure 1c) show that a trapezoidal shape is a simplified model for the real profile of our wedges. In this work, we used the logistic function:

(1) *f* (*x*) = *t* [1 + exp(*k*(*x* − *R*))]^−^^1^

to model the sidewalls, where *k* = 4 tan(θ)/*t* and *x* is the radial coordinate, with origin in the center of the disk. On our samples, this function provides better fits to the profile measurements, as can be seen in Figure 1c.

The resonant structures herein analyzed were intended to be operated in the NIR-visible spectrum. Thus, the chosen dielectrics are:

- silicon nitride (SiN_*x*_) for the core, for its high refractive index and transparency;
- borophosphosilicate glass (BPSG) for the lower cladding, to allow for the fabrication of the vertical coupling scheme, through a planarization technique, as described in [13];
- water for the upper cladding (label-free experiments are actually carried out in buffer solutions, which usually have a refractive index very similar to that of water ±1%).

In order to compare the sensing properties of the commonly-used disk resonators (*i.e.*, those with a vertical sidewall) *versus* wedge resonators, we have run two fabrication processes (the details and method are reported in [14]). These two runs have in common the same plasma-enhanced chemical vapor deposition (PECVD) steps and same lithography masks, but they differ in the etching procedure during the resonator definition. The resulting devices are 350 nm thick, with a radius *R* = 25 µm and *R* = 24 µm for disk and wedge resonators respectively. The disk edge inclination is θ ∼ 85°, while the wedge inclination is θ ∼ 7°. Both resonators are vertically coupled to an integrated silicon oxynitride bus waveguide. Please note that, even if these samples lay on a solid substrate (BPSG), which limits the area available for sensing, a free-standing device could be fabricated for optimal sensing performances, as explained in [13,14].

Laser light with a wavelength near 1550 nm can be coupled in these two structures. Depending on the horizontal displacement of the bus waveguide with respect to the edge of the microresonators, the coupling changes, and therefore, different resonance families can be excited. This parameter was adjusted to achieve a critical coupling to the first and second radial quasi-TE (transverse electric) family modes. We measured the spectral position and quality factor of these resonances as a function of the refractive index of the liquid in contact with the sensor surface. As explained and motivated in Section 3, from these data, we calculated the bulk sensitivity *S* and the bulk figure of merit FOM_b_:

(2) $$S=\frac{\partial \lambda }{\partial n}$$

(3) $$FOM_{b}=\frac{S}{\Gamma }=\frac{QS}{\lambda }$$

where *λ*, Γ and *Q* are the resonance wavelength, linewidth and quality factor, respectively, and *n* is the refractive index of the sensing liquid.

**Figure 2:**
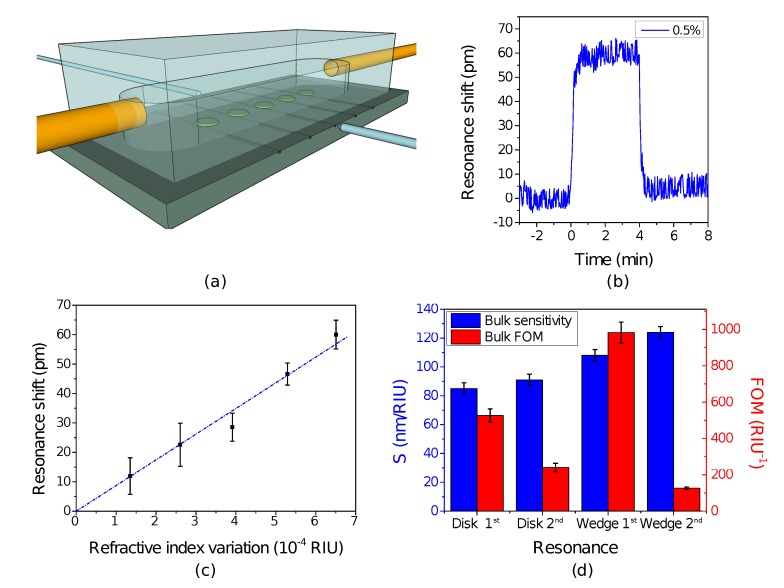
(**a**) 3D model of the experimental apparatus. The waveguides on the photonic chip are accessed with lens-tapered optical fibers. A PDMS flow cell is fixed above the sensor surface, and the liquid is delivered via quartz capillaries; (**b**) Resonance shift temporal evolution during the injection of a small volume of glucose-water solution (concentration 0.5% wt/wt, refractive index variation 6.5× 10^−^^4^ RIU, refractive index unit). The probed resonance comes from the first radial TE (transverse electric) mode of a disk resonator; Experimental data showing the linear relation between resonance wavelength and the bulk refractive index of water-glucose solutions for the first radial TE mode of a disk resonator; Bulk sensitivity and figure of merit measured on the first and second TE radial-family whispering gallery modes (WGMs) of disk and wedge resonators. We considered resonances with center wavelengths close to 1540 nm.

Since the resonance position of our WGM sensors depends on the temperature, with a measured thermal sensitivity of 15 pm/°C, during the experiments, the temperature of the samples was stabilized within 0.01 °C with a Peltier element. The fluid delivery to the sensor surface is achieved with a syringe pump, operated at a constant flow rate of 5 µL/min, and a simple PDMS flow cell. The volume of the flow cell is about 1 µL, with a cross section area at the sensor position of mm^2^. A simple 3D sketch representing the WGM sensors, the flow cell, the capillaries and the lens-tapered optical fibers is depicted in Figure 2a. To change the refractive index of the sensing liquid, we prepared several glucose-water solutions, with concentrations spanning from 0% to 0.5% wt/wt, which provide a refractive index variation of up to 6.5× 10^−^^4^ RIU (refractive index unit). The refractive index of these solutions was measured at higher concentrations with an Abbe refractometer and then was extrapolated linearly to the used range. To corroborate these results, we compared them with the data reported in [15]. The solutions were injected as fixed-volume plugs in the stream of water with the use of a Rheodyne injection valve (loop volume 20 µL). Before and during the dispensing, the spectrum of the resonator has been continuously acquired using a swept laser at wavelengths in the range 1530−1560 nm. The spectral position and quality factor of a selected resonance are measured in real-time by fitting a portion of the spectrum with a Lorentzian function.

In Figure 2b,c, we show an example of the measured resonance shifts for a disk resonator exposed to several water-glucose solutions, and we compare the sensing performances of the disk and wedge resonators in Figure 2d. We notice that, compared to the commonly-used first radial resonance of the disk resonator (*S* = 85 ± 4 nm/RIU), the bulk sensitivity is enhanced by either using the second radial family (*S* = 91 ± 4 nm/RIU) or reducing the inclination (*S* = 108 ± 4 nm/RIU). This is reasonable, since both choices have the effect of reducing the confinement of the WGM and, therefore, of increasing the interaction of the mode with the sensing liquid. However, the WGMs of the second radial family exhibit lower quality factors. In fact, we measured a quality factor *Q*_disk_*_, _*_2_ = 4100 ± 200 and *Q*_wedge_*_, _*_2_ = 1580 ± 30 for the second radial families of disk and wedge resonators, respectively, in comparison with *Q*_disk_*_, _*_1_ = 9600 ± 200 and *Q*_wedge_*_, _*_1_ = 14, 100 ± 300 for the first radial families. By simulating the contributions to the optical losses, as will be explained in the next section, we found that radiative losses are responsible for the lower quality factor of the second radial family modes. For these reasons, the highest bulk FOM is achieved by the wedge resonator’s first radial WGM family. Compared to the disk geometry, which exhibits a FOM_disk_ = 530 ± 40 RIU^−^^1^, the wedge microresonator performs almost twice better, with a FOM_wedge_ = 980 ± 60 RIU^−^^1^.

We need to remark here on a few details about quality factors:

(1) The high-quality factor of the wedge resonator, measured close to the critical coupling (intrinsic quality factor *Q*_0_∼28,000), is not limited by surface scattering, but rather by material absorption. In the adopted spectral region, in fact, silicon nitride has an absorption coefficient in the order of α ∼ 1 cm^−^^1^ [16,17], and water has an absorption coefficient in the order of α ∼ 10 cm^−^^1^ [18]. The same wedge resonators, when dried and exposed only to air, exhibit intrinsic quality factors in excess of *Q*_0_ ≳ 60,000. This is evidence of the limitations imposed by the material absorption, which could be circumvented by changing the operating wavelength, as suggested elsewhere [19].
(2) On the contrary, the lower quality factor that the disk resonator exhibits cannot be explained in terms of material losses or radiation losses. The disk resonator, in fact, has very similar dimensions, the same materials as the wedge one and a lower confinement factor in the water (main source of absorption). The reason for the worse quality is the higher roughness on the sidewall surface of the disk resonator, which is defined via a dry etching instead of a wet etching [9,14]. For a fair comparison between disk and wedge resonators, we would ideally need to compare structures with different geometries and fabricated with similar etching processes.

The results here reported highlight the superior performances (FOM) of sensors defined via an isotropic wet etching step. However, due to the lack of resonator samples with different thickness, it is not clear whether the effect of the sidewall inclination generally improves the sensing or if the same (or better) performances could be obtained with different geometries. To answer this question in a more general way, we decided to analyze the influence of the geometry of the wedge resonator on the sensing performances by means of numerical simulations. This model is also helpful for finding optimized geometrical parameters to achieve the highest figure of merit.

## 3. Problem Analysis and Sensor Design

The problem of the optimization of the sensor can be addressed by different strategies. For example, in a recent paper the author’s aim was to maximize the sensitivity to analyte while minimizing the thermal sensitivity [20]. A different approach is used in [21], where the optimal parameters for minimizing the analyte detection limit are extensively studied starting from the measurement of the amplitude, position and linewidth of the resonance. In this paper, we use an approach similar to that of [22], whereby we analyze both the sensitivity and quality factor of the supported resonant modes as a function of the geometrical parameters of the resonator.

By using a perturbative approach [23,24], the sensitivity *S_V__,__i_* to a variation of the refractive index in the domain *V* and corresponding to the *i*-th WGM with resonance wavelength *λ_i_* can be calculated from the optical mode profile **E***_i_*(**r**) as:

(4) $$S_{V,i}=\frac{\partial \lambda_{i}}{\partial n_{V}}=\frac{\lambda_{i}}{n_{V}}\frac{{∭}_{V}\epsilon (r){\left|E_{i}(r)\right|}^{2}d^{3}r}{{∭}_{\infty }\epsilon (r){\left|E_{i}(r)\right|}^{2}d^{3}r}$$

Here, the two integrals are evaluated in the sensing volume and in the whole model, respectively, and *n*_*V*_ is the unperturbed refractive index of the homogeneous medium contained in *V* . In the same way, in both integrals, the dielectric constant *E*(**r**) and the electric field **E***i*(**r**) are meant to be unperturbed, *i.e.*, when no analyte is present. As can be seen, the sensitivity density $\left(\frac{\lambda_{i}}{n_{V}}\epsilon (r){\left|E_{i}(r)\right|}^{2}/{∭}_{\infty }\epsilon (r){\left|E_{i}(r)\right|}^{2}d^{3}r\right)$ is proportional to the electric field energy density $\frac{1}{2}\epsilon {\left|E\right|}^{2}$. Due to the complex geometrical structure, finite element method (FEM) modeling of the resonator has been used to obtain the mode profiles **E***_i_*(**r**). Since the coupling region between the resonator and bus waveguide is small compared to the WGM size, we assume that the mode profile in the cavity is not significantly altered by the presence of the waveguide, at least far from the coupling region. Thus, our model excludes the waveguide from the simulations. The advantage of this choice is that the cylindrical symmetry of the structure is not broken, and a simple and fast axisymmetric 2.5D simulation can be performed.

When light is confined in a waveguide or in a cavity, it is quite common to obtain evanescent tails, which extend from the surface of the structure for tens to hundreds of nanometers [25,26]. When used as label-free sensors, the volume of interaction between the analyte and the WGM evanescent field is

very small and mainly limited by the thickness of the layer of the captured analyte. In the case of nanometric-sized molecules (like proteins), this means that most of the evanescent tail is unperturbed and does not contribute to the signal. For a fair comparison between different structures, it is very helpful to introduce the superficial sensitivity *σ_A, i_* defined as:

(5) $$\sigma_{A,i}=\frac{\lambda_{i}}{n_{V}}\frac{\iint_{A}\epsilon (r){\left|E_{i}(r)\right|}^{2}d^{2}r}{{∭}_{\infty }\epsilon (r){\left|E_{i}(r)\right|}^{2}d^{3}r}=\frac{\partial^{2}\lambda_{i}}{\partial t_{V}\partial n_{V}}\approx \frac{S_{V,i}}{t_{V}}$$

where *A* is the area of a thin layer of volume *V* and thickness *t*_*V*_ placed on the surface of the sensor. The approximation in Equation (5) is valid for layers *V* much thinner than the extension of the evanescent field (*t_V_* ⪅ 20 nm). Please note the double partial derivative in Equation (5); this is different from the definition usually adopted *∂λ_i_*/*∂t_V_* (see, for example, [22]), which is not independent from the refractive index of the analyte to be sensed in the volume *V* (denser analytes will produce higher fictitious sensitivities). The definition that we adopted only depends on the unperturbed refractive index *n_V_* of the bulk above the sensor area and can be used for direct comparison between different structures or models. To calculate the quality factor, in our model, we account for radiative losses, absorption losses and coupling losses. The first ones are modeled implementing perfect matched layers (PML) at the boundaries of the simulation [27]; the second ones are added in both water and core material as the imaginary part of the refractive index, while the last ones are simply considered by dividing the quality factor by two (critical coupling regime). Assuming that the resonators are defined through a wet etching process, which ensures the best surface quality, in our model, we neglected scattering losses, according to the experimental results discussed at the end of Section 2.

To evaluate the bulk and superficial sensing performances and to choose the optimal set of parameters, we use two figures of merit (FOM) calculated from the sensitivities and quality factors of every resonance:

(6a) $${\mathrm{FOM}}_{b}=\frac{S_{V,i}}{\Gamma_{i}}=\frac{Q_{i}S_{V,i}}{\lambda_{i}}$$

(6b) $${\mathrm{FOM}}_{s}=\frac{\sigma_{A,i}}{\Gamma_{i}}=\frac{Q_{i}\sigma_{A,i}}{\lambda_{i}}$$

From now on, all of the subscripts *i* used to label the different modes are dropped for simplicity of reading, and without any additional specification, *V* and *A* are meant to be the whole sensing volume/area above the parts of the sensor exposed to analytes.

This definition of the bulk figure of merit is equivalent to that used in surface plasmon resonance (SPR) label-free sensors [28]. With this figure of merit, it is possible to directly compare the performances of sensors exploiting different techniques. A table that reviews the FOM of WGM resonators, photonic crystals (PhC) and SPR sensors is reported in [29]. In addition, we also introduce the superficial figure of merit FOM_s_, which in our opinion is a more convenient and fairer index to compare the performances of surface sensors, used, for example, in label-free detection.

For what concerns the WGM sensor, it is common practice to compare their performances in terms of their limit of detection (LOD). This parameter, however, depends also on the features of the apparatus employed in the experiment, like the laser source wavelength uncertainty or the temperature stability. On the contrary, the figure of merit here defined depends only on the WGM resonator, which is the object of our study. If the resolution of the resonance wavelength measurement could be considered limited exclusively by the resonance linewidth itself, one can still estimate the achievable LOD as:

(7) $${\mathrm{LOD}}_{b/s}=\frac{1}{\eta }\frac{1}{{\mathrm{FOM}}_{b/s}}$$

where *η* accounts for an enhanced resolution given by a proper resonance fitting procedure. This parameter *η* depends on experimental details and usually lays in the range 10∼100 [30].

## 4. Wedge Geometry Optimization

Using a commercial FEM solver (Comsol Multiphysics), we solved the electric field eigenfunctions supported by the structure shown in Figure 1. We varied the value of the wedge inclination from θ = 2° to θ ∼ 90 °, and the wedge thickness from *t* = 200 nm to *t* = 500 nm. For every solution, we calculated the bulk and superficial sensitivity, *S* and *σ*, the quality factor, *Q*, and the two figures of merit, FOM_b_*_/_*_s_. The other geometrical parameters were kept fixed, and their values are reported in Table 1.

**Table 1:** Parameters used in the optimization study. BPSG, borophosphosilicate glass.

Parameter	Value	Description
*R*	24 µm	resonator radius
λ	∼1540 nm	resonance wavelength
*n*_water_	1.32	water refractive index (real part)
*k*_water_	1 × 10^−4^	water refractive index (imaginary part) [18,31]
*n*_SiN_	1.99	SiN*_x_* refractive index (real part)
*k*_SiN_	5 × 10^−5^	SiN*_x_* refractive index (imaginary part) [17,31]
*n*_BPSG_	1.46	BPSG refractive index (real part)
*k*_BPSG_	0	BPSG refractive index (imaginary part)
*t_V_*	5 nm	auxiliary layer thickness (for *σ*)

**Figure 3:**
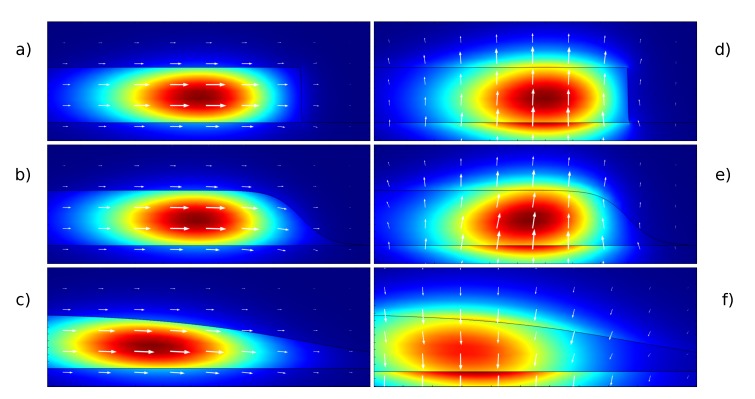
Electric field energy density for the first radial transverse electric, TE, (**a**,**b**,**c**) and transverse magnetic, TM, (**d**,**e**,**f**) modes for wedge angles of 89 (**a**,**d**), 45 (**b**,**e**) and 10 (**c**,**f**) degrees. The white arrows depict the orientation of the electric field. The resonator’s thickness is *t* = 400 nm.

Figure 3 shows the first radial TE and TM (transverse magnetic) modes for three different wedge angles and the same wedge thickness *t* = 400 nm. The difference in the confinement and distribution of the electric field is evident, particularly when comparing the two polarizations. Slightly more difficult to notice, but very interesting, is the fact that the mode reaches its highest confinement for intermediate wedge angles (roughly between 30° and 60°, depending on the other parameters). For greater angles, the field extends mainly from the top surface of the resonator, while for smaller angles, the field is affected by the area above the external sidewall.

**Figure 4:**
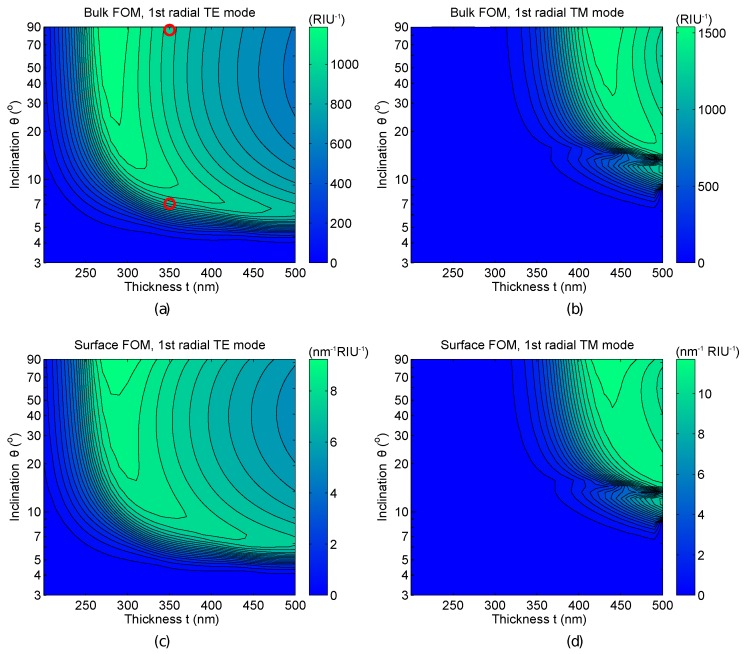
Figure or merit (FOM) analysis performed on the first radial TE (**a**,**c**) and TM (**b**,**d**) modes, as a function of the wedge inclination θ and thickness *t*. The plots report the bulk (**a**,**b**) and superficial (**c**,**d**) figure of merit FOM_b_*_/_*_s_. The red circles in (**a**) label the geometries of the samples characterized in Section 2. Notice that the simulated bulk FOM of the disk geometry is actually slightly higher than the FOM of the wedge geometry. The discrepancy between experimental data and the simulated values is justified by the higher scattering losses of the disk, as thoroughly explained in the text.

In Figure 4, we report the 2D contour plots summarizing the optimization analysis of the bulk and surface FOM for the first radial TE and TM modes. 

For what concerns the bulk sensing (the sensor used as a refractometer for liquids), it is interesting to notice that a wide set of parameters can be used to achieve good performances. Once the thickness has been optimized (*i.e.*, *t* ∼ 280 nm for TE polarization and *t* ∼ 430 nm for TM polarization), the value of the inclination has little impact on the bulk FOM, at least for θ ≳ 40°. In this configuration, we get FOM_b_ ≳ 1200/RIU for TE polarization and FOM_b_ ≳ 1600/RIU for TM polarization. Conversely, the choice of the optimal set of parameters for a sensor used for surface sensing (e.g., a label-free biosensor) is much more strict. The best FOM is achieved with a vertical wedge angle (*i.e.*, a disk resonator) both for TE (FOM_s_ ≳ 9.2/nm RIU) and TM polarization (FOM_s_ ≳ 11.7/nm RIU). We should point out here that it is not possible to create vertical sidewalls with wet etching. However, wedge resonators with an inclination θ ∼ 30° have already been reported [9], and we think that inclinations of at least θ ∼ 40° could be feasible. Thus, even if this analysis is purely theoretical, it still gives very useful guidelines to realize optimized wedge resonators, suggesting the use of the highest feasible inclination and indicating the best thickness depending on θ. 

As expected, the analysis also confirms that the TM polarization can provide higher FOM if the resonator is properly optimized. For bulk sensing, the enhancement can be higher than 30%, while for surface sensing, the enhancement can exceed 20%.

Regarding the experimental results of Section 2, the first observation is that the thickness of the wedge and disk resonators was not optimal for sensing applications. In Figure 4a, the red circles label the parameters of the measured sensors on the bulk FOM map. One can realize that the disk should have been 70 nm thinner, while the wedge should have been 80 nm thicker.

**Figure 5:**
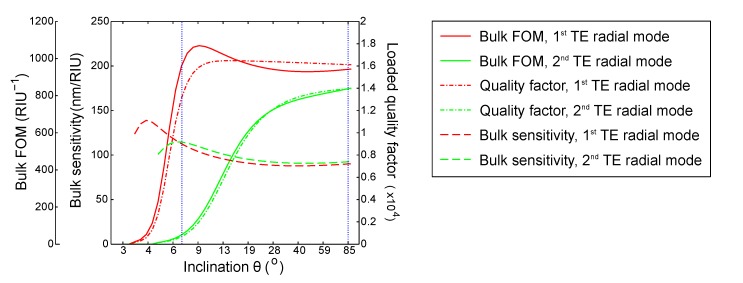
Quality factor (dashed-dot line), bulk sensitivity (dashed line) and bulk FOM (continuous line) for the first (red) and second (green) TE radial modes. The vertical blue lines are markers for the parameters of the fabricated sensors (wedge and disk resonators). The resonator thickness is *t* = 350 nm.

To compare the experimental results with the simulations, Figure 5 reports the bulk FOM together with the bulk sensitivity and quality factor for the first two radial TE modes of a resonator with *t* = 350 nm. For this thickness, the two aforementioned TE modes exhibit the highest FOM_b_, as also observed experimentally.

The figure qualitatively confirms the experimental results: for this thickness, the wedge resonator can provide a higher bulk figure of merit with respect to a disk resonator. Similarly, the bulk sensitivity is higher for θ = 7° than for θ = 85° and even higher for the second mode in both structures. However, the accuracy of the quality factor for the disk in the simulation is poor: in our model, we did not take into account the scattering losses due to the roughness of the resonator. In particular, the disk resonator was defined through a dry etching process (instead of the assumed wet etching), which is known to lead to a higher final roughness, as already pointed out in Section 2. This can explain why the real quality factor, as well as the bulk FOM are actually lower than the simulated ones in the case θ = 85°. The same does not apply to the case of the bulk sensitivity, since *S*_*b*_ does not depend on the roughness of the structure.

The results of our analysis show that: first, the optimal wedge inclination is the highest one achievable and, secondly, the control on the resonator thickness is crucial for the realization of optimized sensors. The use of vertical coupling is thus indicated to have this freedom in the fabrication without imposing limitations on the geometry of the bus waveguide [13]. This technique, in fact, relies on two deposition and lithography steps, where the thickness of both deposited layers can be changed independently according to the results of the optimization analysis.

## 5. Discussion

In this work, we tested the effectiveness of vertically-coupled integrated wedge resonators for refractometric sensing applications, with a particular focus on label-free biosensing. These devices are very appealing because of the very high quality factor that they can exhibit. Nevertheless, the quality factor is not the only concern when analyzing the device performances. The figures of merit of interest for sensing applications are also affected by the field overlap with the analyte.

We experimentally verified that under certain conditions, a wedge resonator can perform better compared to a disk resonator with similar dimensions. At the same time, however, we also showed by means of numerical analysis that the optimal design for a wedge sensor requires the highest achievable wedge inclination θ and the proper choice of the thickness.

In the modeling, we used two figures of merit (bulk and surface) that permit a proper and fair comparison between sensors with different geometries, even between sensors relying on different physical principles (e.g., SPR or PhC). The bulk FOM shows that our structures perform at least one order of magnitude better than SPR devices for bulk refractometric sensing, which are in the order of FOM_b_ ≈ 100/RIU [29]. In addition, we introduced the surface FOM, which can be used to compare different structures with special focus on surface sensing (like the label-free method).

To summarize, the use of wedge resonators in place of conventional disk resonators is suggested. The isotropic wet etching step is necessary to obtain better surface quality, but care has to be taken in order to keep the inclination as high as possible, ideally better if θ ≳ 40 °, at least for resonators similar to the ones described in Table 1. The analysis here presented shows that the optimization of the resonator thickness is critical to achieve the best performances. In this regard, the use of vertical coupling has emerged as particularly appealing, because it gives control of the resonator thickness and geometry without posing limitations to the bus waveguide. In addition, the vertical coupling could allow the realization of free-standing sensors.

## References

[1] Ciminelli C., Campanella C.M., Dell’Olio F., Campanella C.E., Armenise M.N. (2013). Label-free optical resonant sensors for biochemical applications. Prog. Quantum Electron..

[2] Serpengüzel A., Griffel G., Arnold S. (1995). Excitation of resonances of microspheres on an optical fiber. Opt. Lett..

[3] Vollmer F., Braun D., Libchaber A., Khoshsima M., Teraoka I., Arnold S. (2002). Protein detection by optical shift of a resonant microcavity. Appl. Phys. Lett..

[4] De Vos K., Girones J., Claes T., De Koninck Y., Popelka S., Schacht E., Baets R., Bienstman P. (2009). Multiplexed Antibody Detection With an Array of Silicon-on-Insulator Microring Resonators. IEEE Photonics J..

[5] Washburn A.L., Luchansky M.S., Bowman A.L., Bailey R.C. (2010). Quantitative, label-free detection of five protein biomarkers using multiplexed arrays of silicon photonic microring resonators. Anal. Chem..

[6] Gaathon O., Culic-Viskota J., Mihnev M., Teraoka I., Arnold S. (2006). Enhancing sensitivity of a whispering gallery mode biosensor by subwavelength confinement. Appl. Phys. Lett..

[7] Zamora V., Díez A., Andrés M.V., Gimeno B. (2007). Refractometric sensor based on whispering-gallery modes of thin capillarie. Opt. Express.

[8] Armani D.K., Kippenberg T.J., Spillane S.M., Vahala K.J. (2003). Ultra-high-Q toroid microcavity on a chip. Nature.

[9] Lee H., Chen T., Li J., Yang K.Y., Jeon S., Painter O., Vahala K.J. (2012). Chemically etched ultrahigh-Q wedge-resonator on a silicon chip. Nat. Photonics.

[10] Sedlmeir F., Zeltner R., Leuchs G., Schwefel H.G. (2014). High-Q MgF2 whispering gallery mode resonators for refractometric sensing in aqueous environment. Opt. Express.

[11] Iqbal M., Gleeson M.A., Spaugh B., Tybor F., Gunn W.G., Hochberg M., Baehr-Jones T., Bailey R.C., Gunn L.C. (2010). Label-Free Biosensor Arrays Based on Silicon Ring Resonators and High-Speed Optical Scanning Instrumentation. IEEE J. Sel. Top. Quantum Electron..

[12] Pasquardini L., Berneschi S., Barucci A., Cosi F., Dallapiccola R., Insinna M., Lunelli L., Conti G.N., Pederzolli C., Salvadori S. (2013). Whispering gallery mode aptasensors for detection of blood proteins. J. Biophotonics.

[13] Ghulinyan M., Guider R., Pucker G., Pavesi L. (2011). Monolithic Whispering-Gallery Mode Resonators With Vertically Coupled Integrated Bus Waveguides. IEEE Photonics Technol. Lett..

[14] Ramiro-Manzano F., Prtljaga N., Pavesi L., Pucker G., Ghulinyan M. (2012). A fully integrated high-Q Whispering-Gallery Wedge Resonator. Opt. Express.

[15] bin Mat Yunus W.M., bin Abdul Rahman A. (1988). Refractive index of solutions at high concentrations. Appl. Opt..

[16] Germann R., Salemink H.W.M., Beyeler R., Bona G.L., Horst F., Massarek I., Offrein B.J. (2000). Silicon Oxynitride Layers for Optical Waveguide Applications. J. Electrochem. Soc..

[17] Kischkat J., Peters S., Gruska B., Semtsiv M., Chashnikova M., Klinkmüller M., Fedosenko O., Machulik S., Aleksandrova A., Monastyrskyi G. (2012). Mid-infrared optical properties of thin films of aluminum oxide, titanium dioxide, silicon dioxide, aluminum nitride, and silicon nitride. Appl. Opt..

[18] Kou L., Labrie D., Chylek P. (1993). Refractive indices of water and ice in the 0.65- to 2.5-µm spectral range. Appl. Opt..

[19] Armani A.M., Armani D.K., Min B., Vahala K.J., Spillane S.M. (2005). Ultra-high-Q microcavity operation in *H*_2_*O* and *D*_2_*O*. Appl. Phys. Lett..

[20] Delezoide C., Ledoux-Rak I., Nguyen C.T. (2014). General approach for the sensitivity analysis and optimization of integrated optical evanescent-wave sensors. J. Opt. Soc. Am. B.

[21] Foreman M.R., Jin W.L., Vollmer F. (2014). Optimizing detection limits in whispering gallery mode biosensing. Opt. express.

[22] Ciminelli C., Dell’Olio F., Conteduca D., Campanella C., Armenise M. (2014). High performance SOI microring resonator for biochemical sensing. Opt. Laser Technol..

[23] Johnson S., Ibanescu M., Skorobogatiy M., Weisberg O., Joannopoulos J., Fink Y. (2002). Perturbation theory for Maxwell’s equations with shifting material boundaries. Phys. Rev. E.

[24] Teraoka I., Arnold S., Vollmer F. (2003). Perturbation approach to resonance shifts of whispering-gallery modes in a dielectric microsphere as a probe of a surrounding medium. J. Opt. Soc. Am. B.

[25] Rigo E., Aparicio F.J., Vanacharla M.R., Larcheri S., Guider R., Han B., Pucker G., Pavesi L. (2013). Evanescent-field excitation and collection approach for waveguide based photonic luminescent biosensors. Appl. Phys. B.

[26] Aparicio F.J., Froner E., Rigo E., Gandolfi D., Scarpa M., Han B., Ghulinyan M., Pucker G., Pavesi L. (2014). Silicon oxynitride waveguides as evanescent-field-based fluorescent biosensors. J. Phys. D: Appl. Phys..

[27] Cheema M.I., Kirk A.G. (2013). Accurate determination of the quality factor and tunneling distance of axisymmetric resonators for biosensing applications. Opt. express.

[28] Shen Y., Zhou J., Liu T., Tao Y., Jiang R., Liu M., Xiao G., Zhu J., Zhou Z.K., Wang X., Jin C., Wang J. (2013). Plasmonic gold mushroom arrays with refractive index sensing figures of merit approaching the theoretical limit. Nat. Commun..

[29] Huang L., Tian H., Yang D., Zhou J., Liu Q., Zhang P., Ji Y. (2014). Optimization of figure of merit in label-free biochemical sensors by designing a ring defect coupled resonator. Opt. Commun..

[30] Vollmer F., Yang L. (2012). Review Label-free detection with high-Q microcavities: A review of biosensing mechanisms for integrated devices. Nanophotonics.

[31] Polyanskiy M.N. (2008). Refractive index database. http://refractiveindex.info.

